# Supplementary material for: In silico EsxG EsxH rational epitope selection: Candidate epitopes for vaccine design against pulmonary tuberculosis
Source: PLoS One. 2023 Apr 20;18(4):e0284264. doi: 10.1371/journal.pone.0284264 (PMC10118122; doi:10.1371/journal.pone.0284264)
Supplement: S1 Table — 49 HLA-DR alleles were considered to locate promiscuous binding regions. The output consists of a list of nonameric epitopes capable of binding into a given HLA-DR allele. (DOCX) [file pone.0284264.s001.docx]

| Alleles | Epitopes (EsxG) | Alleles | Epitopes (EsxH) | Alleles | Epitopes (EsxH) |
| --- | --- | --- | --- | --- | --- |
| DRB1 0102  DRB1 0305  DRB1 0306  DRB1 0307  DRB1 0308  DRB1 0311  DRB1 0401  DRB1 0402  DRB1 0404  DRB1 0408  DRB1 0421  DRB1 0423  DRB1 0426  DRB1 1107 | ^11^ LVASQSAFA ^19^ | DRB1 0102  DRB1 0301  DRB1 0305  DRB1 0306  DRB1 0307  DRB1 0308  DRB1 0309  DRB1 0311  DRB1 0402  DRB1 0404  DRB1 0405  DRB1 0408  DRB1 0410  DRB1 0423  DRB1 0801  DRB1 0802  DRB1 0804  DRB1 0806  DRB1 0813  DRB1 0817  DRB1 1101  DRB1 1102  DRB1 1104  DRB1 1106  DRB1 1107  DRB1 1114  DRB1 1120  DRB1 1121  DRB1 1128  DRB1 1301  DRB1 1302  DRB1 1304  DRB1 1305  DRB1 1307  DRB1 1311  DRB1 1321  DRB1 1322  DRB1 1323  DRB1 1327  DRB1 1328  DRB1 1501  DRB1 1502  DRB1 1506 | ^66^ VRAYHAMSS ^74^ | DRB1 0301  DRB1 0305  DRB1 0306  DRB1 0307  DRB1 0308  DRB1 0309  DRB1 0311  DRB1 0402  DRB1 0404  DRB1 0423  DRB1 0801  DRB1 0802  DRB1 0804  DRB1 0806  DRB1 0813  DRB1 1101  DRB1 1102  DRB1 1104  DRB1 1106  DRB1 1107  DRB1 1114  DRB1 1120  DRB1 1121  DRB1 1128  DRB1 1301  DRB1 1302  DRB1 1304  DRB1 1305  DRB1 1307  DRB1 1311  DRB1 1321  DRB1 1322  DRB1 1323  DRB1 1327  DRB1 1328 | ^65^ LVRAYHAMS ^73^ |
| DRB1 0101  DRB1 0102  DRB1 0305  DRB1 0401  DRB1 0405  DRB1 0408  DRB1 0426  DRB1 0801  DRB1 0802  DRB1 0813  DRB1 1101  DRB1 1120  DRB1 1302  DRB1 1307  DRB1 1323 | ^83^ YVAADAAAA ^91^ | DRB1 0402  DRB1 0801  DRB1 0802  DRB1 0803  DRB1 0804  DRB1 0806  DRB1 0813  DRB1 0817  DRB1 1102  DRB1 1121  DRB1 1322 | ^83^ MMARDTAEA ^90^ | DRB1 0801  DRB1 0802  DRB1 0804  DRB1 0813  DRB1 0817  DRB1 1501  DRB1 1502  DRB1 1506 | ^5^ MYNYPAMLG ^13^ |
| DRB1 0301  DRB1 0306  DRB1 0307  DRB1 0308  DRB1 0309  DRB1 0311  DRB1 1107 | ^84^ VAADAAAAS ^92^ | DRB1 0305  DRB1 0306  DRB1 0307  DRB1 0308  DRB1 0311  DRB1 1107 | ^84^ MARDTAEAA _92_ | DRB1 0101  DRB1 0401  DRB1 0402  DRB1 0405  DRB1 0408  DRB1 0421  DRB1 0426 | ^54^ WQAQWNQAM ^62^ |
| DRB1 0101  DRB1 0102  DRB1 0801  DRB1 1321  DRB5 0101  DRB5 0105 | ^58^ FVAAAAKVN ^66^ | DRB1 0306  DRB1 0307  DRB1 0308  DRB1 0311 | ^81^ MAMMARDTA ^89^ | DRB1 0405  DRB1 0408  DRB1 0410  DRB1 0421 | ^69^ YHAMSSTHE ^77^ |
| DRB1 1102  DRB1 1107  DRB1 1121  DRB1 1322  DRB1 1323 | ^24^ LMRHTIGQA ^32^ | DRB1 0101  DRB1 0102  DRB1 0423 | ^25^ LQSLGAEIA ^33^ | DRB1 0101  DRB1 0102  DRB1 0806 | ^4^ IMYNYPAML ^12^ |
| DRB1 0423 | ^8^ IPQLVASQS ^16^ | DRB1 0305  DRB1 0309 | ^43^ WQGDTGITY ^51^ | DRB5 0101  DRB5 0105 | ^8^ YPAMLGHAG ^16^ |
|  |  | DRB1 0401 | ^28^ LGAEIAVEQ ^36^ | DRB1 0401 | ^32^ IAVEQAALQ ^40^ |
