# Supplementary figures and images for: In silico EsxG EsxH rational epitope selection: Candidate epitopes for vaccine design against pulmonary tuberculosis
Source: PLoS One. 2023 Apr 20;18(4):e0284264. doi: 10.1371/journal.pone.0284264 (PMC10118122; doi:10.1371/journal.pone.0284264)

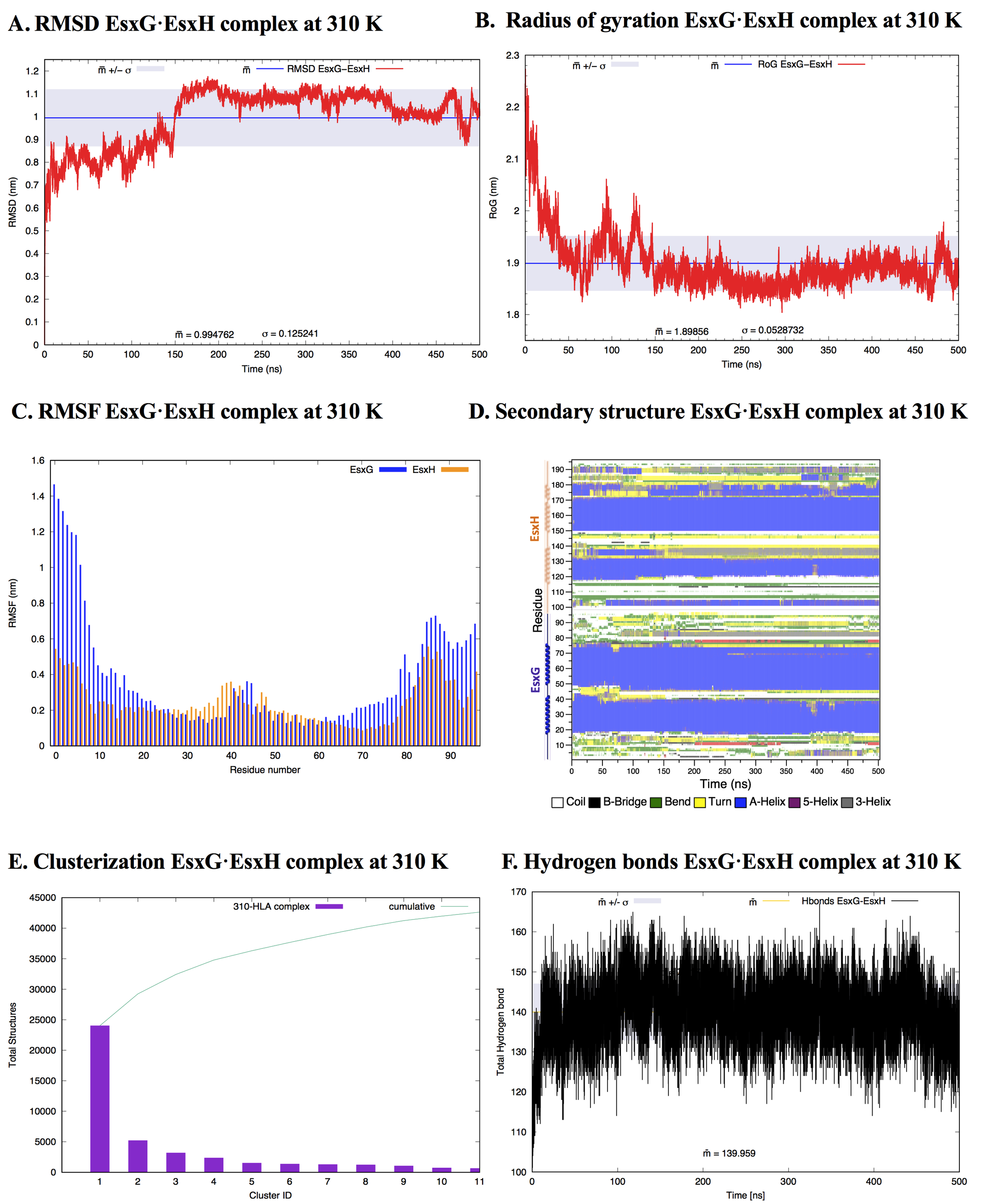

Supplement: S1 Fig — A. Root Mean Square Deviation (RMSD) plot from EsxG EsxH complex at 310 K. Red line represents the RMSD value during 500 ns of simulation. Blue line represents the RMSD average. Shaded bar represents standard deviation. B. Radius of Gyration (RoG) plot from EsxG EsxH complex at 310 K. Red line stand for RoG value during 500 ns of simulation. Blue line stands for the RoG average. Shaded bar represents standard deviation. C. Root Mean Square Fluctuation (RMSF) plot from EsxG EsxH complex at 310 K. X axis corresponds to residue number. The blue color depicts the EsxG monomer. The orange color depicts the EsxH monomer. D. Secondary structure plot from EsxG EsxH complex at 310 K. Residues 1 to 97 represents EsxG monomer, residues 98 to 194 represents EsxH monomer. E. Clusterization plot from EsxG EsxH complex at 310 K. X axis stand for the cluster ID. Y axis stand for the number of structures in the cluster. F. Hydrogen bonds plot from EsxG EsxH complex at 310 K. Black line stand for total hydrogen bonds during 500 ns of simulation. (TIF) [file pone.0284264.s002.tif]

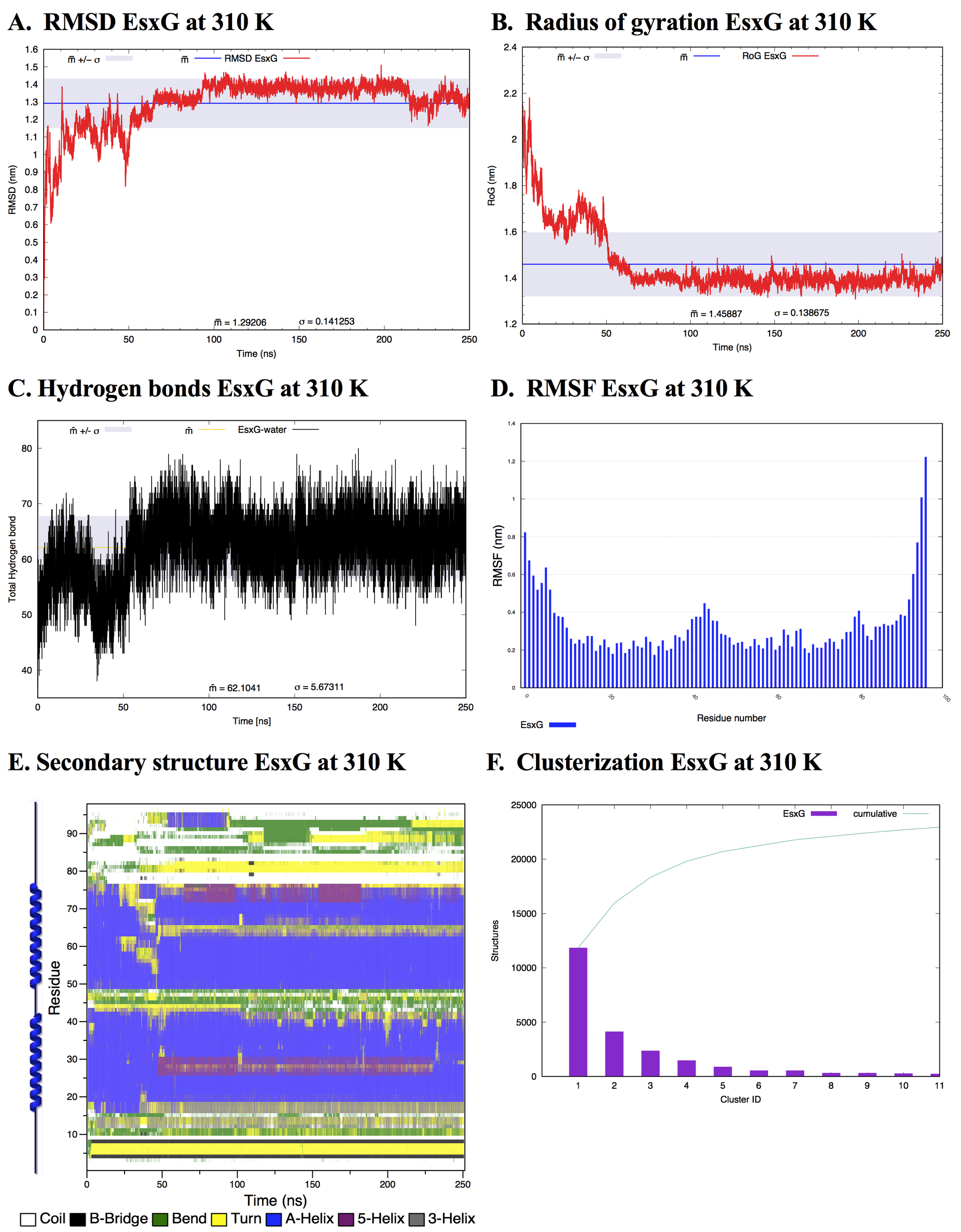

Supplement: S2 Fig — A. Root Mean Square Deviation (RMSD) plot from EsxG monomer at 310 K. Red line depict the RMSD value during 250 ns of simulation. Blue line depicts the RMSD average. Shaded bar represents standard deviation. B. Radius of Gyration (RoG) plot from EsxG monomer at 310 K. Red line represents RoG value during 250 ns of simulation. Blue line represents the RoG average. Shaded bar represents standard deviation. C. Hydrogen bonds plot from EsxG monomer at 310 K. Black line stand for total hydrogen bonds during 250 ns of simulation. D. Root Mean Square Fluctuation (RMSF) plot from EsxG monomer at 310 K. X axis corresponds to residue number. E. Secondary structure plot from EsxG monomer at 310 K. X axis corresponds to residue number. F. Clusterization plot from EsxG monomer at 310 K. X axis represents the cluster ID. Y axis represents the number of structures. (TIF) [file pone.0284264.s003.tif]

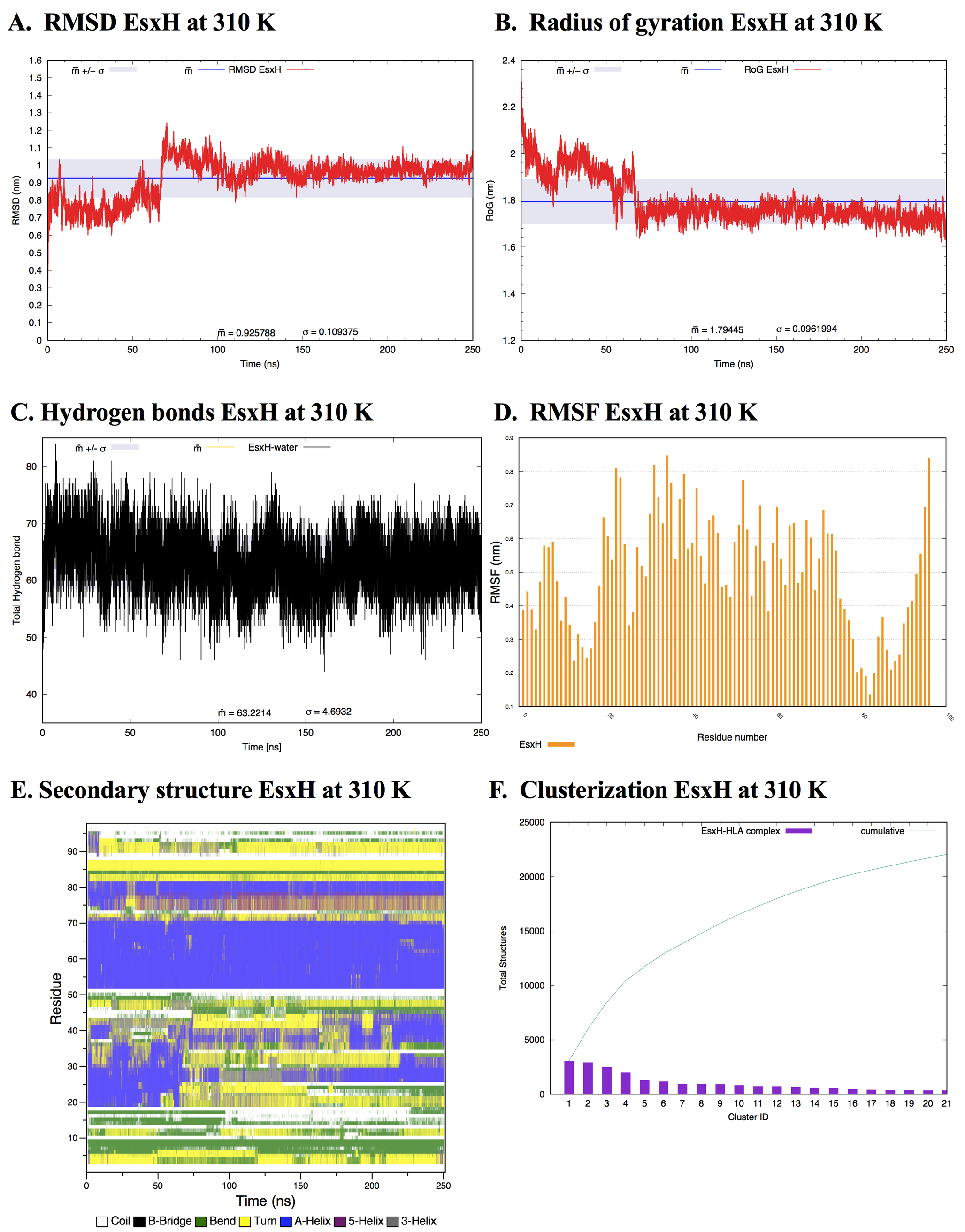

Supplement: S3 Fig — A. Root Mean Square Deviation (RMSD) plot from EsxH monomer at 310 K. Red line depict the RMSD value during 250 ns of simulation. Blue line depicts the RMSD average. Shaded bar represents standard deviation. B. Radius of Gyration (RoG) plot from EsxH monomer at 310 K. Red line represents RoG value during 250 ns of simulation. Blue line represents the RoG average. Shaded bar represents standard deviation. C. Hydrogen bonds plot from EsxH monomer at 310 K. Black line represents total hydrogen bonds during 250 ns of simulation. D. Root Mean Square Fluctuation (RMSF) plot from EsxH monomer at 310 K. X axis corresponds to residue number. E. Secondary structure plot from EsxH monomer at 310 K. X axis corresponds to residue number. F. Clusterization plot from EsxH monomer at 310 K. X axis represents the cluster ID. Y axis represents the number of structures. (TIF) [file pone.0284264.s004.tif]

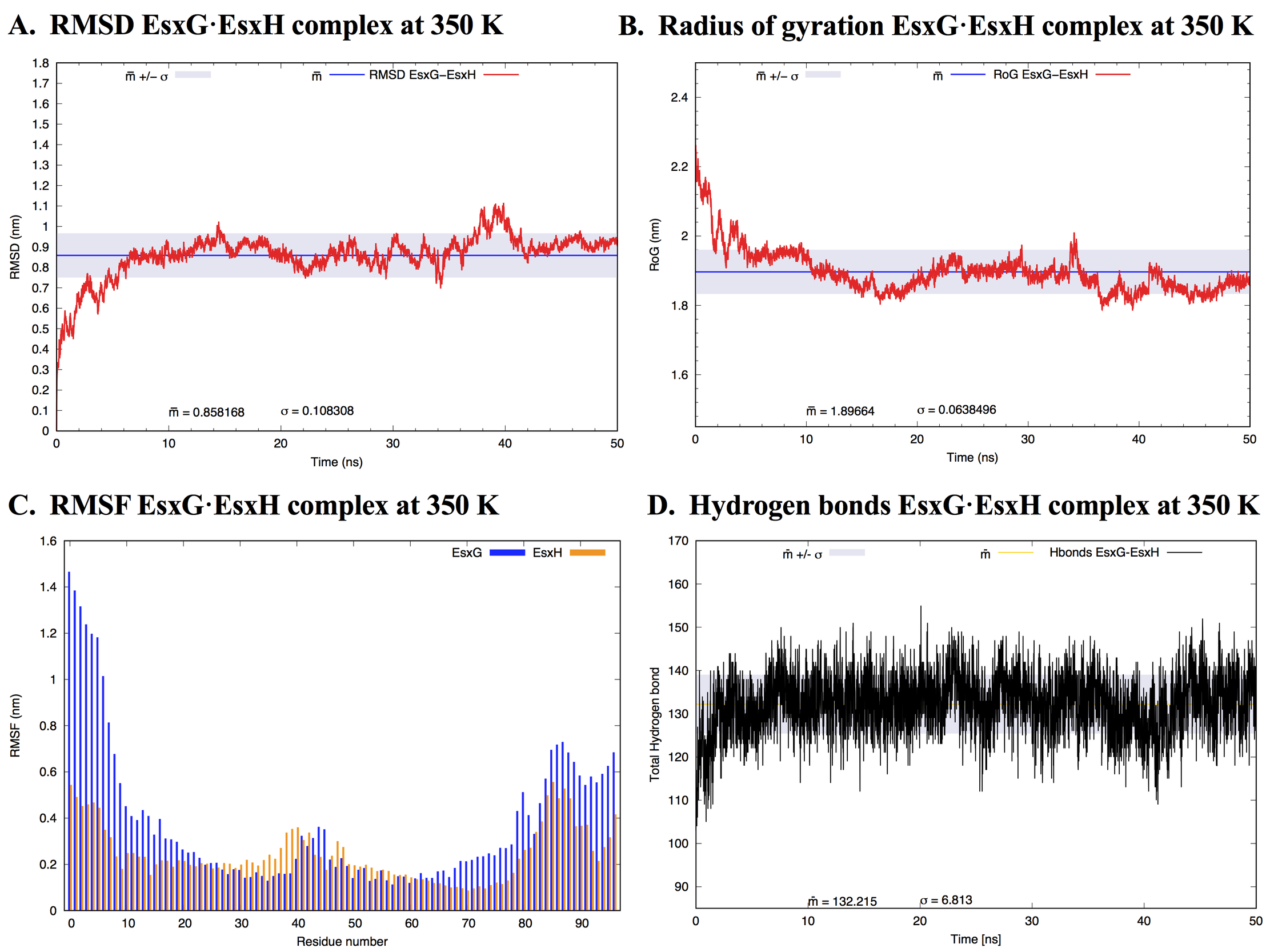

Supplement: S4 Fig — A. Root Mean Square Deviation (RMSD) plot from EsxG EsxH complex at 350 K. Red line stand for the RMSD value during 50 ns of simulation. Blue line stands for the RMSD average. Shaded bar represents standard deviation. B. Radius of Gyration (RoG) plot from EsxG EsxH complex at 350 K. Red line depict RoG value during 50 ns of simulation. Blue line depicts the RoG average. Shaded bar represents standard deviation. C. Root Mean Square Fluctuation (RMSF) plot from EsxG EsxH complex at 350 K. X axis corresponds to residue number. The blue color depicts the EsxG monomer. The orange color represents the EsxH monomer. D. Hydrogen bonds plot from EsxG EsxH complex at 350 K. Black line represents total hydrogen bonds during 50 ns of simulation. (TIF) [file pone.0284264.s005.tif]

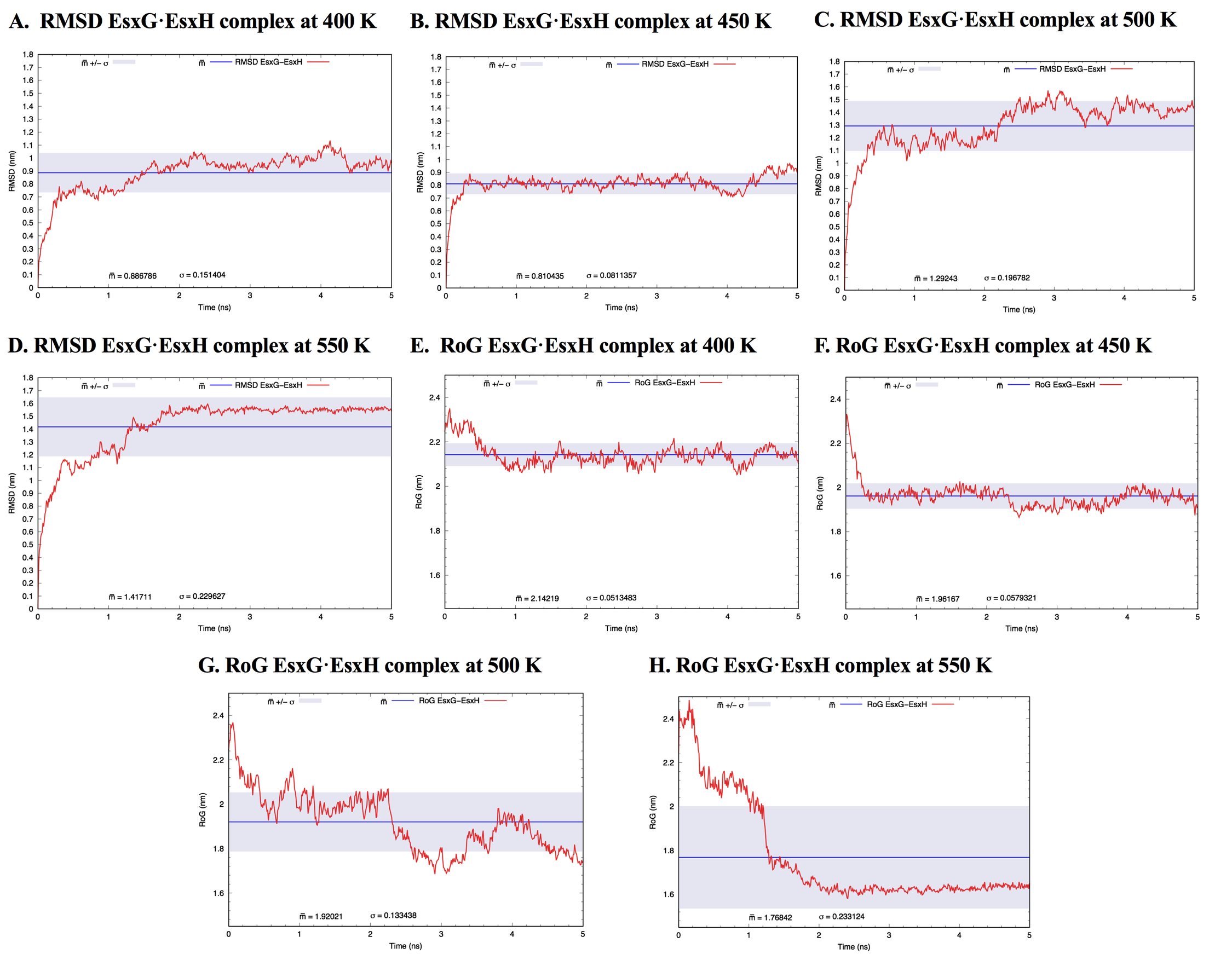

Supplement: S5 Fig — A. Root Mean Square Deviation (RMSD) plot from EsxG EsxH complex at 400 K. Red line represents the RMSD value during 5 ns of simulation. Blue line represents the RMSD average. Shaded bar represents standard deviation. B. Root Mean Square Deviation (RMSD) plot from EsxG EsxH complex at 450 K. Red line stand for the RMSD value during 5 ns of simulation. Blue line stands for the RMSD average. Shaded bar stands for standard deviation. C. Root Mean Square Deviation (RMSD) plot from EsxG EsxH complex at 500 K. Red line depict the RMSD value during 5 ns of simulation. Blue line depicts the RMSD average. Shaded bar depicts standard deviation. D. Root Mean Square Deviation (RMSD) plot from EsxG EsxH complex at 550 K. Red line represents the RMSD value during 5 ns of simulation. Blue line represents the RMSD average. Shaded bar represents standard deviation. E. Radius of Gyration (RoG) plot from EsxG EsxH complex at 400 K. Red line stand for RoG value during 5 ns of simulation. Blue line stands for the RoG average. Shaded bar stands for standard deviation. F. Radius of Gyration (RoG) plot from EsxG EsxH complex at 450 K. Red line represents RoG value during 5 ns of simulation. Blue line represents the RoG average. Shaded bar represents standard deviation. G. Radius of Gyration (RoG) plot from EsxG EsxH complex at 500 K. Red line depict RoG value during 5 ns of simulation. Blue line depicts the RoG average. Shaded bar represents standard deviation. H. Radius of Gyration (RoG) plot from EsxG EsxH complex at 550 K. Red line represents RoG value during 5 ns of simulation. Blue line represents the RoG average. Shaded bar represents standard deviation. (TIF) [file pone.0284264.s006.tif]

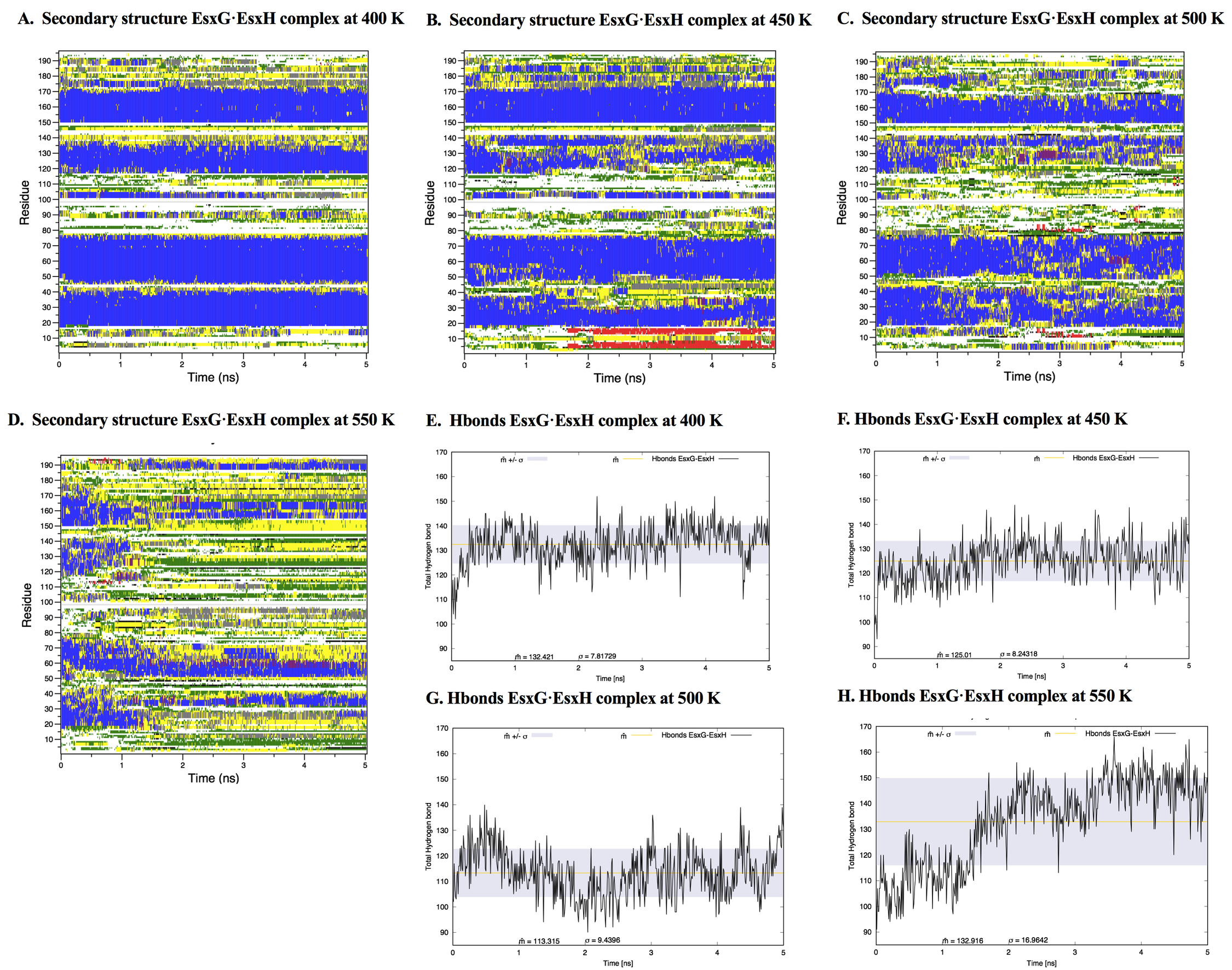

Supplement: S6 Fig — A. Secondary structure plot from EsxG EsxH complex at 400 K. Residues 1 to 97 depict EsxG monomer, residues 98 to 194 depict EsxH monomer. B. Secondary structure plot from EsxG EsxH complex at 450 K. Residues 1 to 97 depict EsxG monomer, residues 98 to 194 represents EsxH monomer. C. Secondary structure plot from EsxG EsxH complex at 500 K. Residues 1 to 97 stand for EsxG monomer, residues 98 to 194 stand for EsxH monomer. D. Secondary structure plot from EsxG EsxH complex at 550 K. Residues 1 to 97 represents EsxG monomer, residues 98 to 194 represents EsxH monomer. E. Hydrogen bonds plot from EsxG EsxH complex at 400 K. Black line depict total hydrogen bonds during 5 ns of simulation. F. Hydrogen bonds plot from EsxG EsxH complex at 450 K. Black line depict total hydrogen bonds during 5 ns of simulation. G. Hydrogen bonds plot from EsxG EsxH complex at 500 K. Black line stand for total hydrogen bonds during 5 ns of simulation. H. Hydrogen bonds plot from EsxG EsxH complex at 550 K. Black line stand for total hydrogen bonds during 5 ns of simulation. (TIF) [file pone.0284264.s007.tif]

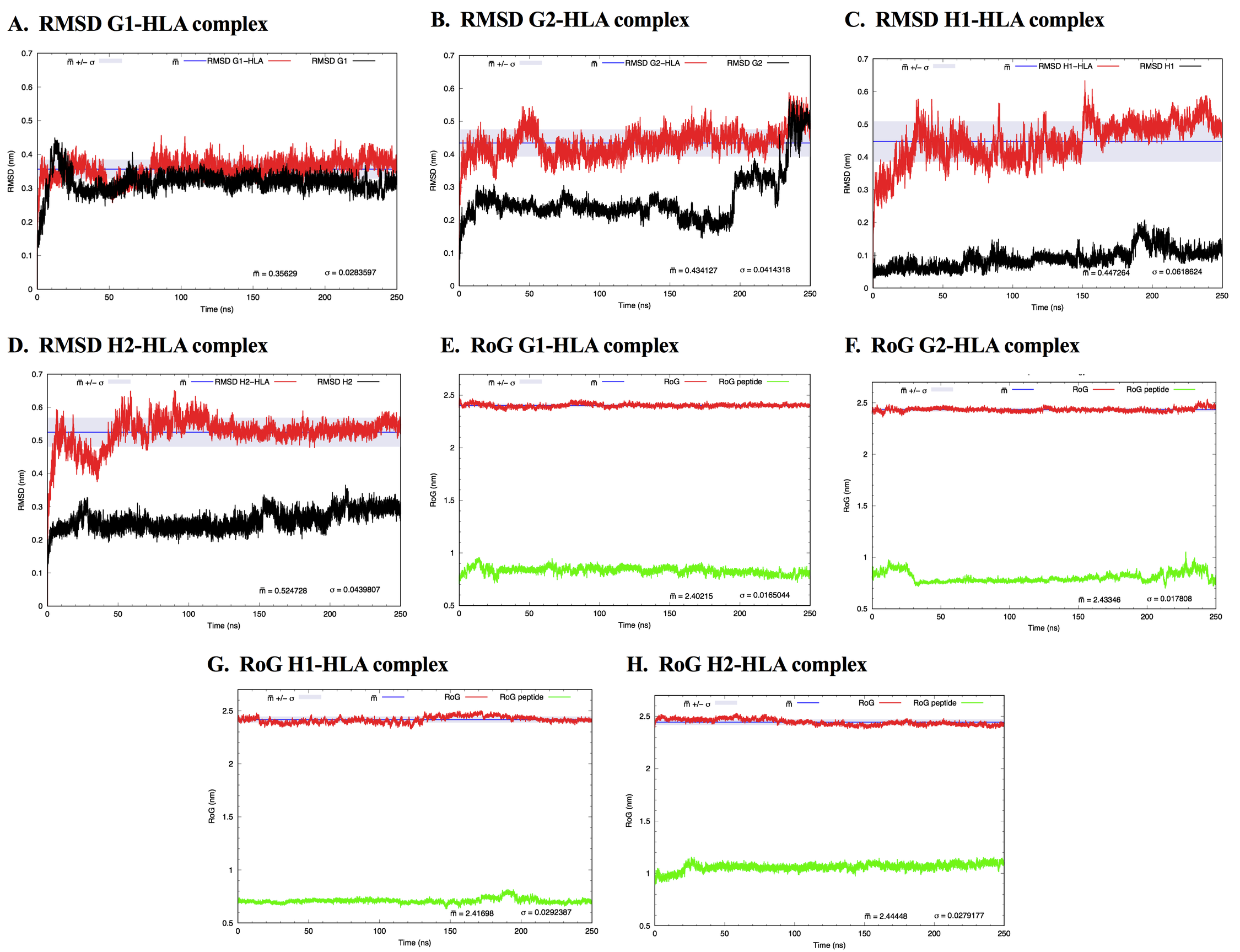

Supplement: S7 Fig — A. Root Mean Square Deviation (RMSD) plot from G1 HLA complex at 310 K. Red line represents the RMSD value from the G1-HLA complex during 250 ns of simulation. Black line represents the RMSD value from the G1 epitope in solution during 250 ns of simulation. Blue line represents the RMSD average. Shaded bar represents standard deviation. B. Root Mean Square Deviation (RMSD) plot from G2 HLA complex at 310 K. Red line depict the RMSD value from the G2-HLA complex during 250 ns of simulation. Black line depicts the RMSD value from the G2 epitope in solution during 250 ns of simulation. Blue line depicts the RMSD average. Shaded bar represents standard deviation. C. Root Mean Square Deviation (RMSD) plot from H1 HLA complex at 310 K. Red line stand for the RMSD value from the H1-HLA complex during 250 ns of simulation. Black line stands for the RMSD value from the H1 epitope in solution during 250 ns of simulation. Blue line stands for the RMSD average. Shaded bar stands for standard deviation. D. Root Mean Square Deviation (RMSD) plot from H2 HLA complex at 310 K. Red line represents the RMSD value from the H2-HLA complex during 250 ns of simulation. Black line represents the RMSD value from the H2 epitope in solution during 250 ns of simulation. Blue line represents the RMSD average. Shaded bar represents standard deviation. E. Root Mean Square Deviation (RMSD) plot from G1 HLA complex at 310 K. Red line depict the RMSD value from the G1-HLA complex during 250 ns of simulation. Green line depicts the RMSD value from the G1 epitope in solution during 250 ns of simulation. Blue line depicts the RMSD average. Shaded bar represents standard deviation. F. Root Mean Square Deviation (RMSD) plot from G1 HLA complex at 310 K. Red line stand for the RMSD value from the G2-HLA complex during 250 ns of simulation. Green line stands for the RMSD value from the G2 epitope in solution during 250 ns of simulation. Blue line stands for the RMSD average. Shaded bar represents standard [file pone.0284264.s008.tif]

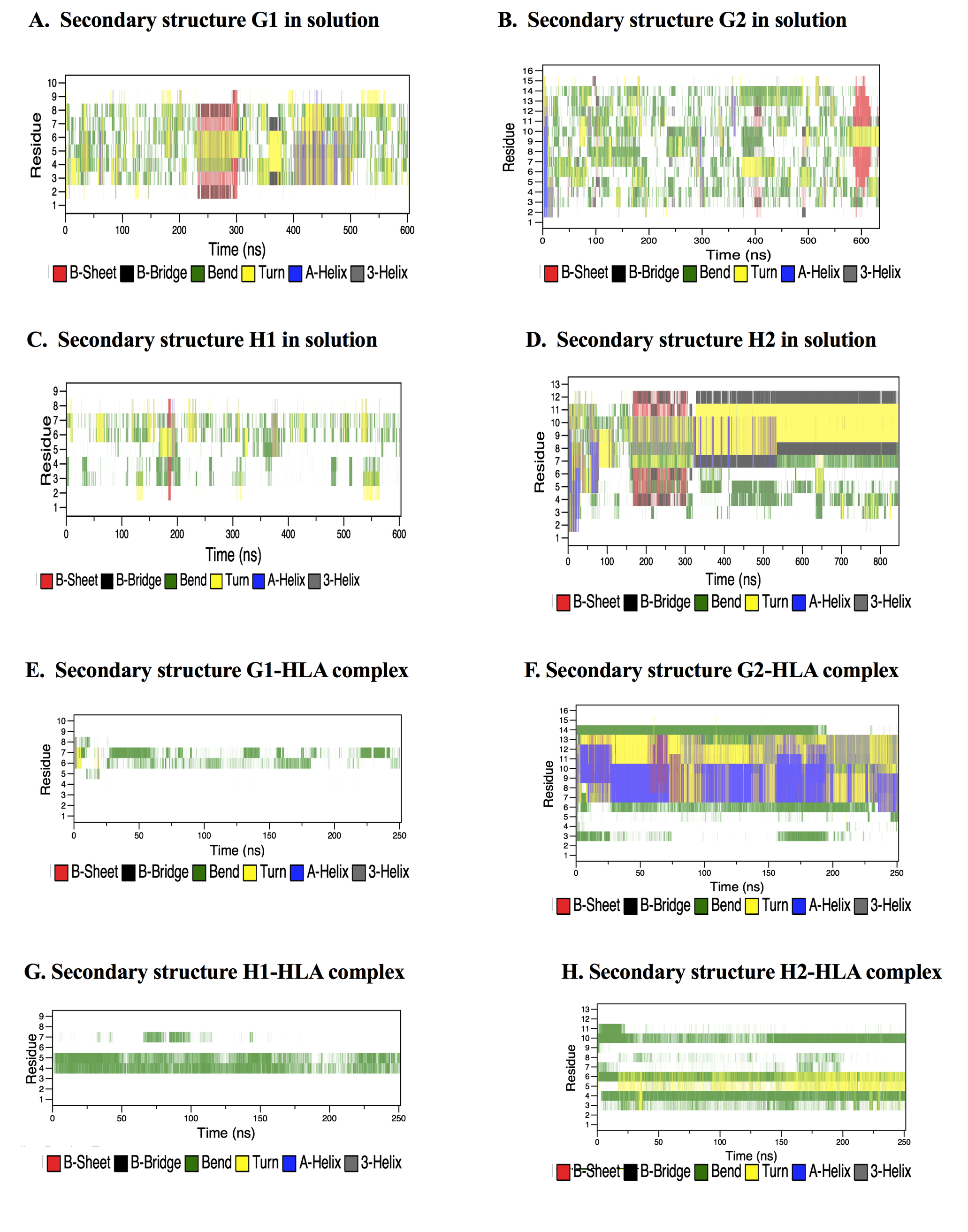

Supplement: S8 Fig — A. Secondary structure plot from G1 epitope in solution at 310 K. Residues 1 to 9 stand for the G1 epitope LVASQSAFA. B. Secondary structure plot from G2 epitope in solution at 310 K. Residues 1 to 16 stand for the G2 epitope FQAAHARFVAAAAKVN. C. Secondary structure plot from H1 epitope in solution at 310 K. Residues 1 to 9 represents the H1 epitope MYNYPAMLG. D. Secondary structure plot from H2 epitope in solution at 310 K. Residues 1 to 13 represents the H2 epitope LVRAYHAMSSTHE. E. Secondary structure plot from G1 epitope in HLA at 310 K. Residues 1 to 9 depict the G1 epitope LVASQSAFA. F. Secondary structure plot from G2 epitope in HLA at 310 K. Residues 1 to 16 depict the G2 epitope FQAAHARFVAAAAKVN. G. Secondary structure plot from H1 epitope in HLA at 310 K. Residues 1 to 9 depict the H1 epitope MYNYPAMLG. H. Secondary structure plot from H2 epitope in HLA at 310 K. Residues 1 to 13 depict the H2 epitope LVRAYHAMSSTHE. (TIF) [file pone.0284264.s009.tif]

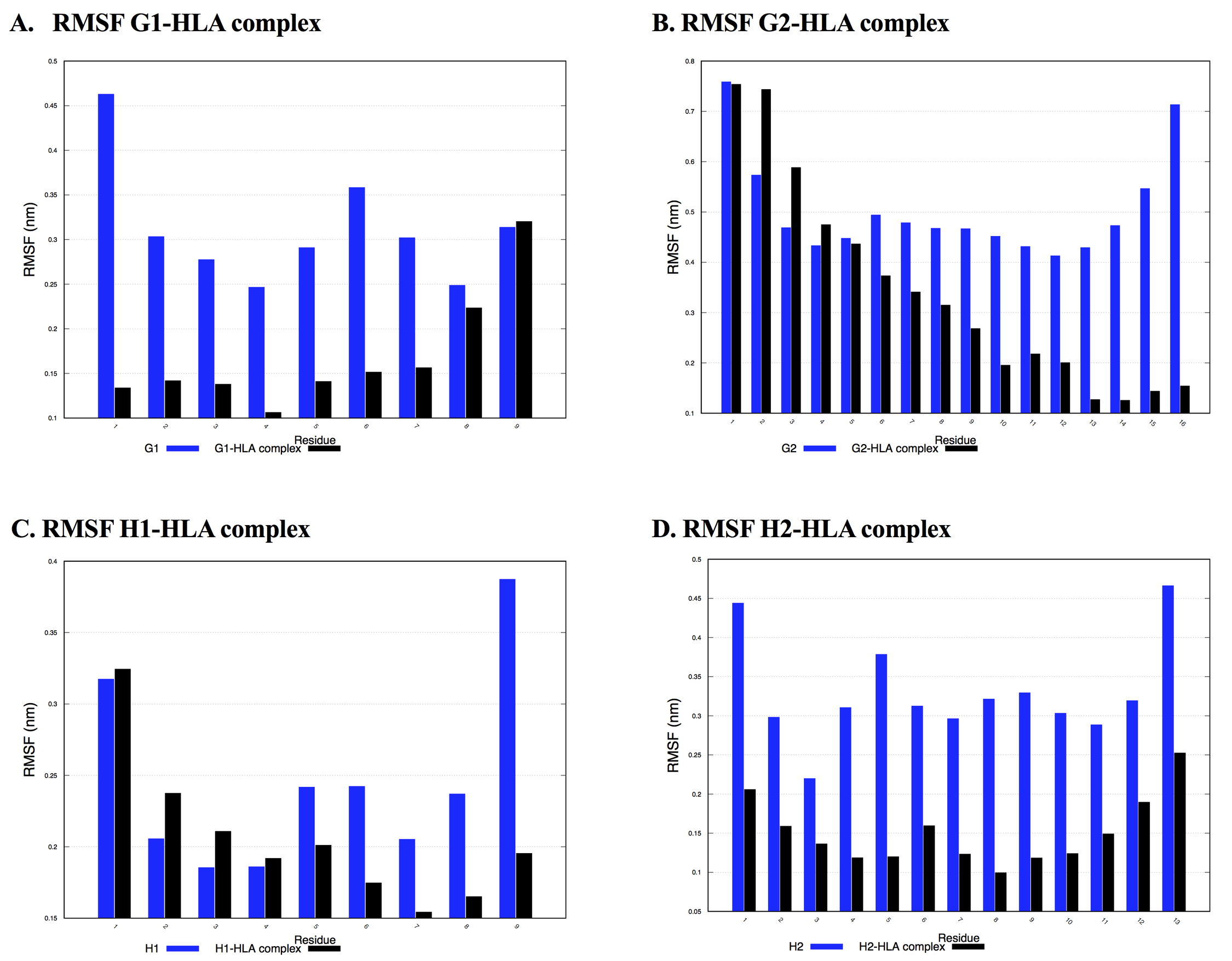

Supplement: S9 Fig — A. Root Mean Square Fluctuation (RMSF) plot from G1 epitope in solution, and in HLA complex at 310 K. X axis corresponds to residue number, residues 1 to 9 stand for the G1 epitope LVASQSAFA. Blue color stand for G1 epitope in solution. Black color stand for G1 epitope in complex with HLA. B. Root Mean Square Fluctuation (RMSF) plot from G2 epitope in solution, and in HLA complex at 310 K. X axis corresponds to residue number, residues 1 to 16 depict the G2 epitope FQAAHARFVAAAAKVN. Blue color represents G1 epitope in solution. Black color depicts G1 epitope in complex with HLA. C. Root Mean Square Fluctuation (RMSF) plot from H1 epitope in solution, and in HLA complex at 310 K. X axis corresponds to residue number, residues 1 to 9 depict the H1 epitope MYNYPAMLG. Blue color depicts G1 epitope in solution. Black color depicts G1 epitope in complex with HLA. D. Root Mean Square Fluctuation (RMSF) plot from H2 epitope in solution, and in HLA complex at 310 K. X axis corresponds to residue number, residues 1 to 13 represents the H2 epitope LVRAYHAMSSTHE. Blue color represents G1 epitope in solution. Black color represents G1 epitope in complex with HLA. (TIF) [file pone.0284264.s010.tif]

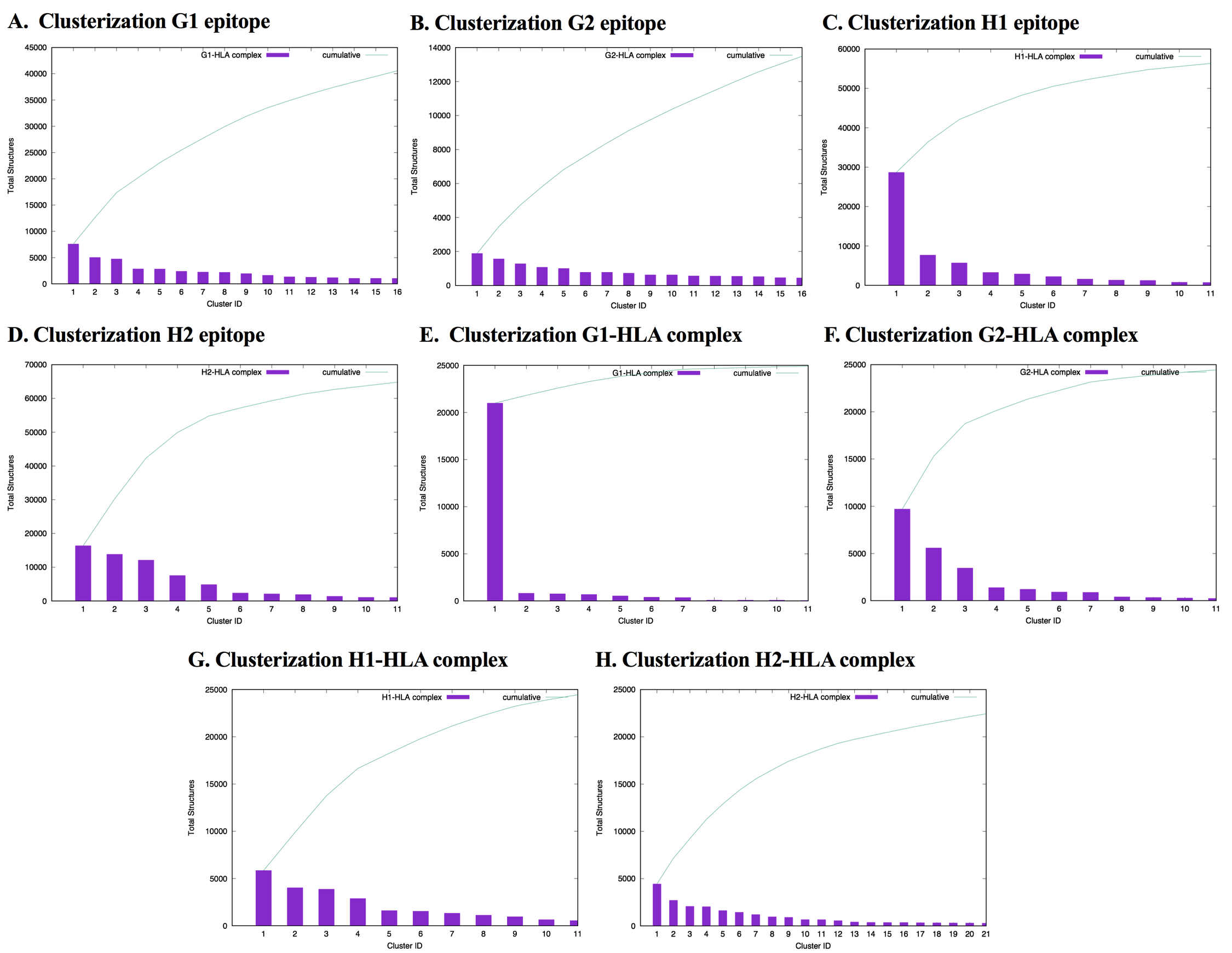

Supplement: S10 Fig — A. Clusterization plot from G1 epitope in solution at 310 K. X axis depict the cluster ID. Y axis depict the number of structures. B. Clusterization plot from G2 epitope in solution at 310 K. X axis represents the cluster ID. Y axis represents the number of structures. C. Clusterization plot from H1 epitope in solution at 310 K. X axis stand for the cluster ID. Y axis stand for the number of structures. D. Clusterization plot from H2 epitope in solution at 310 K. X axis represents the cluster ID. Y axis represents the number of structures. E. Clusterization plot from G1 epitope in HLA at 310 K. X axis depict the cluster ID. Y axis depict the number of structures. F. Clusterization plot from G2 epitope in HLA at 310 K. X axis stand for the cluster ID. Y axis stand for the number of structures. G. Clusterization plot from H1 HLA in solution at 310 K. X axis represents the cluster ID. Y axis represents the number of structures. H. Clusterization plot from H2 epitope in HLA at 310 K. X axis stand for the cluster ID. Y axis stand for the number of structures. (TIF) [file pone.0284264.s011.tif]

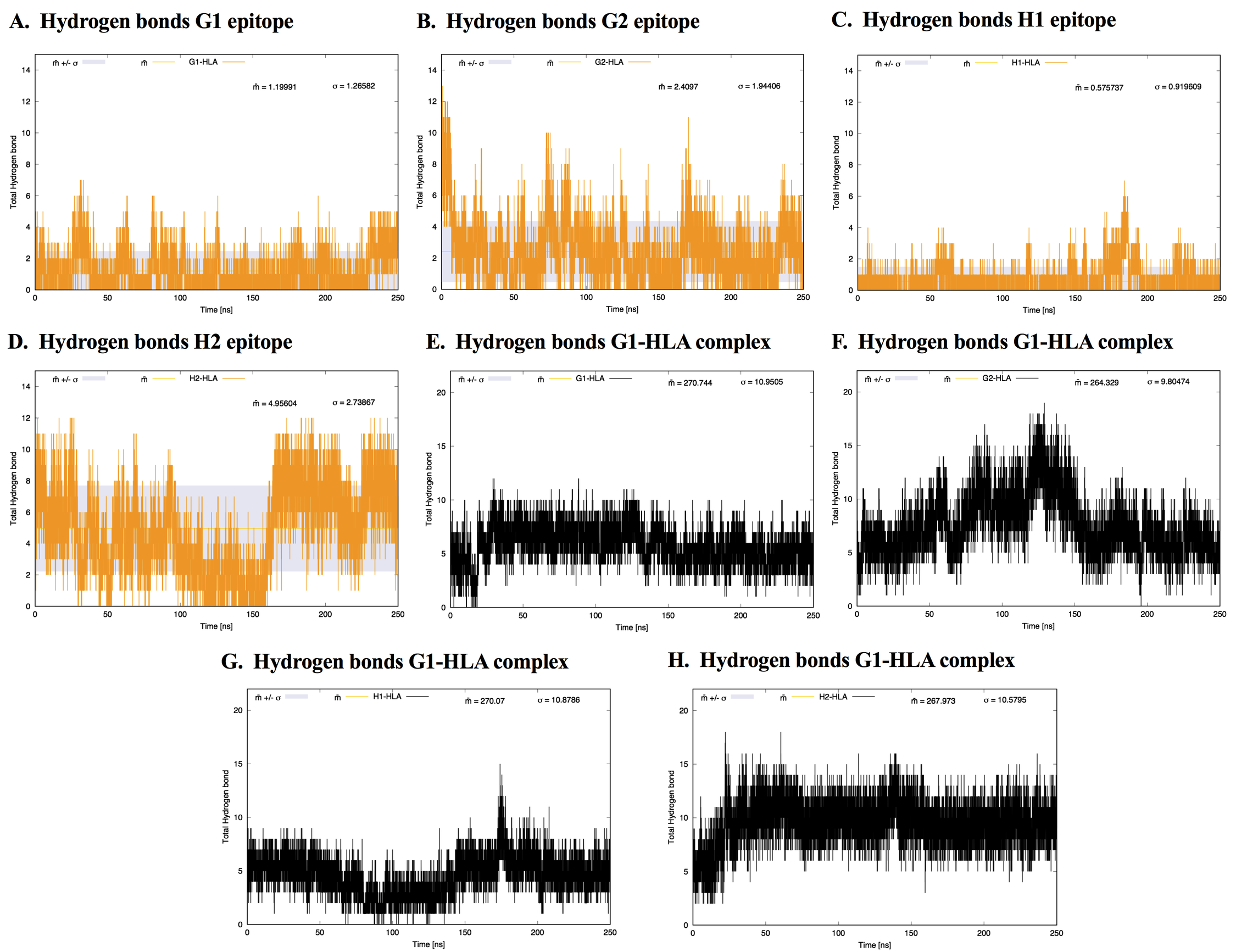

Supplement: S11 Fig — A. Hydrogen bonds plot from G1 in solution at 310 K. Orange line depict total hydrogen bonds during 250 ns of simulation. B. Hydrogen bonds plot from G2 in solution at 310 K. Orange line depict total hydrogen bonds during 250 ns of simulation. C. Hydrogen bonds plot from H1 in solution at 310 K. Orange line represents total hydrogen bonds during 250 ns of simulation. D. Hydrogen bonds plot from H2 in solution at 310 K. Orange line represents total hydrogen bonds during 250 ns of simulation. E. Hydrogen bonds plot from G1 in HLA at 310 K. Black line stand for total hydrogen bonds during 250 ns of simulation. F. Hydrogen bonds plot from G2 in HLA at 310 K. Black line stand for total hydrogen bonds during 250 ns of simulation. G. Hydrogen bonds plot from H1 in HLA at 310 K. Black line represents total hydrogen bonds during 250 ns of simulation. H. Hydrogen bonds plot from H2 in HLA at 310 K. Black line represents total hydrogen bonds during 250 ns of simulation. (TIF) [file pone.0284264.s012.tif]
