# Supplementary material for: In silico EsxG EsxH rational epitope selection: Candidate epitopes for vaccine design against pulmonary tuberculosis
Source: PLoS One. 2023 Apr 20;18(4):e0284264. doi: 10.1371/journal.pone.0284264 (PMC10118122; doi:10.1371/journal.pone.0284264)
Supplement: Attachment — Submitted filename: response_reviewers.pdf [file pone.0284264.s014.pdf]

Reviewer #2: Dear Authors,
it's a bit hard for me to catch the new advancements and revisions to indicate if you have adequately addressed my comments. Could you please provide a new version with highlighted or marked in bold parts concerning my comments?
Thank you.

Author response

Reviewer #2. We regret being unclear in our previous response. In order to make all suggestions easier to understand and read, we are currently underlining and highlighting them in the "Revised Manuscript with Track Changes" file.

On October 25th, we received the very first comments and advice from Reviewers of our submitted work titled “In silico EsxG·EsxH Rational Epitope Selection: Candidate Epitopes for Vaccine Design against Pulmonary Tuberculosis” . In this way, reviewer #2's comments stated:

Reviewer #2: The paper by Martinez-Olivares et al. describes a rational design of epitope-based peptide vaccines by using bioinformatics and structural vaccinology tools, focusing on the testing of EsxG·EsxH complex. The ESX systems from Mycobacterium tuberculosis are very well-known factors for the secretion of highly immunogenic proteins that seem of key importance for bacterial survival and growth. I appreciated the fact that, even if the authors use already investigated tools and approaches, they explore the fusion of EsxG and EsxH proteins as candidate target for TB vaccine development, never tested before in this conjugated version, especially for the presence of EsxH protein.

The pipeline is well described and the results appropriately report the potentiality to be considered as subunit vaccine candidates or conjugated vaccines, even if the validation phase is mandatory for the evaluation of the immunogenicity. The conclusions convey the main points of the manuscript and the methodology is exhaustively described in the Methods section.

I would recommend the authors to address my concerns here below before final pubblication in order to provide to the scientific community this further step in the field of TB vaccine development.
- Inside the Introduction part, the authors should integrate the bibliography related to the rational vaccine design through immunobioinformatics tools and computational chemistry approaches. They report only two references (not so recent) while the literature offers more innovative and updated examples of new vaccine development pipeline such as the one reported in Sunita et al., 2019 (doi:10.1080/21645515.2019.1670035) and some specific examples applied for example to COVID-19 vaccine (doi: 10.1093/bib/bbab403).

Author response

We modified the manuscript in order to update cites about new techniques and immunoinformatics, also we mentioned both cite that Reviewer #2 advised to us. That changes are present in Pag. 2 lines 32 to 38 in the Introduction section of the final Manuscript. It is worth mentioning that references 5, 6, 7, 8, 9, 10 and, 39 were added as recommended by Reviewer #2.

- In Results section, the authors mention the role of the MTB in provoking intracellular infection, justifying the importance of MHC class II epitope prediction. For the sake of completeness, the authors should also report specific results related also to the extracellular infection that Mtb is able to cause and the consequent main immunological actors dynamics involved in this perspective. I would suggest then to insert new plots or piece of evidence in such view, for example focusing on MHC class I epitope prediction.

Author response

We have included the reports that reviewer #2 pointed out. These modifications are present in the final Manuscript, Pag. 10, lines 218 to 224 in the Results section.

- The "Thermal unfolding simulations" is missing the bibliography. Please insert it.

Author response

This reference is placed already in Pag. 7, line 148.

- Even if the Methods are well described, I would suggest the authors to insert inside the manuscript a summary figure reporting all the steps of the rational design approach applied.

Author response

A new figure (Fig 1 in the final manuscript) was made for this proposal. In Pag. 9, lines 195 to 201 contain explanation about that Summary Figure.

Reviewer #3: Please add the full name of" EsxG·EsxH" protein complex in the beginning of the study and then through out the manuscript the abbreviation can be used

Author response

Thank you very much for your comments, we addressed the new change that you suggested to us this time.

We have completed the name of the EsxG·EsxH" protein complex in the abstract section (Pag. 1, lines 3 and 4) and, in the Introduction section, Pag. 3, lines 60 and 61 in the final Manuscript.
